# Surveying biomedical relation extraction: a critical examination of current datasets and the proposal of a new resource

**DOI:** 10.1093/bib/bbae132

**Published:** 2024-04-12

**Authors:** Ming-Siang Huang, Jen-Chieh Han, Pei-Yen Lin, Yu-Ting You, Richard Tzong-Han Tsai, Wen-Lian Hsu

**Affiliations:** Intelligent Agent Systems Laboratory, Department of Computer Science and Information Engineering, Asia University, New Taipei City, Taiwan; National Institute of Cancer Research, National Health Research Institutes, Tainan, Taiwan; Department of Computer Science and Information Engineering, College of Information and Electrical Engineering, Asia University, Taichung, Taiwan; Intelligent Information Service Research Laboratory, Department of Computer Science and Information Engineering, National Central University, Taoyuan, Taiwan; Intelligent Agent Systems Laboratory, Department of Computer Science and Information Engineering, Asia University, New Taipei City, Taiwan; Intelligent Agent Systems Laboratory, Department of Computer Science and Information Engineering, Asia University, New Taipei City, Taiwan; Intelligent Information Service Research Laboratory, Department of Computer Science and Information Engineering, National Central University, Taoyuan, Taiwan; Center for Geographic Information Science, Research Center for Humanities and Social Sciences, Academia Sinica, Taipei, Taiwan; Intelligent Agent Systems Laboratory, Department of Computer Science and Information Engineering, Asia University, New Taipei City, Taiwan; Department of Computer Science and Information Engineering, College of Information and Electrical Engineering, Asia University, Taichung, Taiwan

**Keywords:** relation extraction, protein–protein interaction, natural language processing

## Abstract

Natural language processing (NLP) has become an essential technique in various fields, offering a wide range of possibilities for analyzing data and developing diverse NLP tasks. In the biomedical domain, understanding the complex relationships between compounds and proteins is critical, especially in the context of signal transduction and biochemical pathways. Among these relationships, protein–protein interactions (PPIs) are of particular interest, given their potential to trigger a variety of biological reactions. To improve the ability to predict PPI events, we propose the protein event detection dataset (PEDD), which comprises 6823 abstracts, 39 488 sentences and 182 937 gene pairs. Our PEDD dataset has been utilized in the AI CUP Biomedical Paper Analysis competition, where systems are challenged to predict 12 different relation types. In this paper, we review the state-of-the-art relation extraction research and provide an overview of the PEDD’s compilation process. Furthermore, we present the results of the PPI extraction competition and evaluate several language models’ performances on the PEDD. This paper’s outcomes will provide a valuable roadmap for future studies on protein event detection in NLP. By addressing this critical challenge, we hope to enable breakthroughs in drug discovery and enhance our understanding of the molecular mechanisms underlying various diseases.

## INTRODUCTION

Biomedical natural language processing (BioNLP) has the potential to revolutionize healthcare, enabled by electronic health records (EHR), large biomedical text corpora and machine learning (ML)/NLP techniques. We now have the capability to extract valuable insights from unstructured biomedical text [1], such as EHR [2–4], scientific literature [5, 6] and clinical notes [7, 8]. To make progress in BioNLP, high-quality datasets and experts to build models are indispensable.

**Figure 1:**
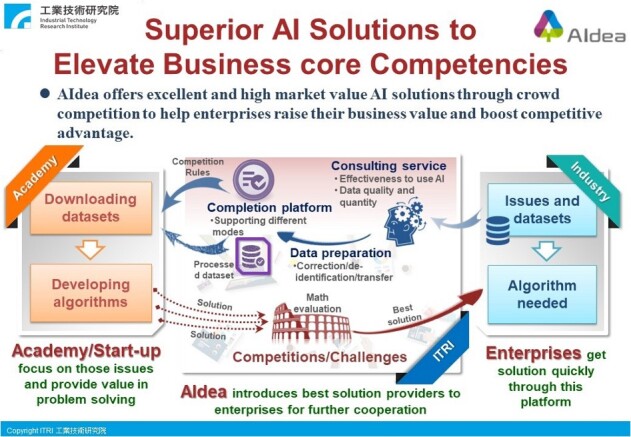
Operation schema of the AIdea platform.

The AI CUP, the abbreviation for the National University Artificial Intelligence Competition initiated by the Ministry of Education in Taiwan, project aims to advance BioNLP by funding research teams to curate datasets and organizing competitions to engage ML developers. In 2018, the AI CUP project secured funding for the annotation of the EBED dataset [9] and organized a biomedical named entity recognition (BNER) competition in the AIdea platform [10] (Figure 1). The competition attracted numerous experts and led to a considerable enhancement in the BNER task.

The extraction of information on molecular pathways and signal transduction from bio-entities to bio-relations is crucial for biologists, allowing them to efficiently retrieve data. Several target pairs and corresponding relation types have been defined in relation datasets, such as the bacteria biotope (BB) task [11, 12], protein–gene interaction [13–17], drug–drug interaction (DDI) [18–20], microRNA-target interaction (MTI), adverse drug effect (ADE) [21] and drug–disease association [22–24]. Additionally, in the context of precision medicine and the enhanced utilization of EHR [2–4], relationships between medication, indication and clinical diagnosis have also gained attention.

In 2019, we received funding from the AI CUP project to propose a protein–protein interaction extraction (PPIE) competition on a new biomedical dataset called the protein event detection dataset (PEDD). The competition attracted 439 participants and raised the level of PPIE further.

In this paper, we will compare existing biomedical relation datasets, present the definitions of the PPIE track and the PEDD information, and elaborate on the filtering process used to ensure credible content. We will also provide statements for every relation type, with representative instances and a simplified classification to reduce complexity. Statistics of the PEDD are reported to demonstrate the distribution profiles of relation types, and the performance of participant systems is presented, along with the strategies they incorporated. This review serves as a case study to clarify model preferences when dealing with similar problems.

## RELATED WORK

This section provides an overview of the current state of research in biological relation extraction, including datasets, target pairs of interest and traditional and popular approaches.

### Overview of RE datasets

This section provides a comprehensive overview of the various datasets and challenges associated with relation extraction (RE) in the biomedical field. We discuss datasets, such as General Language Understanding Evaluation (GLUE), miRTarBase, PREDICT and others, highlighting their importance in identifying complex relationships in biomedical text. Furthermore, we discuss prominent competitions such as BioCreative and BioNLP Shared Task (BioNLP-ST), both of which have played a critical role in text mining technology advancement. The article also discusses challenges associated with event extraction and drug–disease associations, providing insight into the diversity of tasks and datasets in the biomedical domain.

RE uses ML to identify relationships between named entities (NEs) in text. This is often done by training models on prepared datasets with defined scopes. Various datasets have been developed to help progress the field and address the challenge of identifying complex networks of relationships.

Two well-known datasets for RE are GLUE benchmark [25] and miRTarBase [26]. GLUE is provided in the general domain for several relation identification tasks. miRTarBase 9.0 contains 13 389 articles for MTI with 27 172 target genes from 37 species, aiding treatments and drug developments for miRNA-related diseases.

Gottlieb *et al.* [22] designed PREdicting Drug IndiCaTions (PREDICT), an algorithm using the Unified Medical Language System (UMLS) [27] to rank potential drug–disease associations for predicting drug indications. The list contains 183 676 possible associations between 593 drugs from DrugBank [28] and 313 diseases in the Online Mendelian Inheritance in Man (OMIM) database [29], providing reliable support for disease indications or drug repositioning studies.

Yang *et al.* [30] have extracted 3175 side-effect (SE)-disease relationships by combining SE-drug (888 drugs and 584 SEs in SIDER database [31]) and drug–disease (303 drugs and 145 diseases in PharmGKB [32]) relationships. The disease-associated SEs are gathered as training features that formulate the human phenotypic profiles for additional indications of drugs. The Naïve Bayes models can predict indications for 145 diseases after training. Additionally, 4200 clinical molecules from Genego MetaBase serve as indications for 101 disease subsets.

Most biomedical relation datasets adopt MEDLINE, PubMed and PubMed Central (PMC) as major data resources, with clinical texts becoming increasingly important. Datasets are released to encourage research progression. A recent proposal, BioRED [33], integrated individual RE datasets into a comprehensive dataset. Furthermore, BioRED was used in BioCreative VIII Track 1, where participants had to handle various biomedical RE datasets simultaneously, adding both challenge and breadth. It represents the largest-scale application of RE datasets in recent years. However, individual sources still need to be listed in detail. Tables 1 and 2 provide an overview of biomedical relation datasets and challenges.

**Table 1:** Overview of relation extraction challenge datasets

Task	Dataset name	Release	Data source	Scale/data size	Relation objects	Relation types	Reference
BioCreative II-PPI	Dataset for interaction pair subtask (IPS)	2006	PubMed	1098 full-texts (740 for training, 358 for test)	Protein	Binary interaction	[34]
BioCreative II.5	Interaction pairs task (IPT)	2009	FEBS Letters	122 full-texts	Protein	Binary interaction	[35]
2010 i2b2/VA challenge – relation (now is n2c2)	2010 i2b2 dataset	2010	Three health Facilities	871 EMRs (394 for training, 477 for test)	Medical problem, treatment, test concepts	Medical problems-treatments, medical problems-tests, medical problems-other medical problems	[51]
BioNLP-ST 2011	Entity relations (REL)	2011	MEDLINE	1210 abstracts	Gene/protein and the other entity	Protein-component and subunit-complex	[44]
	Epigenetics and post-translational modifications (EPI)	2011	PubMed	1200 abstracts	Protein, event	15 types	[44]
	Genia event extraction (GE)	2011	MEDLINE, PMC	1210 abstracts; 14 full-texts	Protein, event	9 types	[42]
	Infectious diseases (ID)	2011	PMC	30 full-texts	Protein, chemical, organism, two-component-system and regulon-operon	10 types	[44]
DDIExtraction 2011 challenge	DrugDDI corpus	2011	DrugBank	5806 sentences/579 texts	Drug	Binary interaction	[18]
BioNLP-ST 2013	Genia event extraction (GE)	2013	PMC	34 full-texts	Protein, event	13 types	[46]
	Cancer genetics (CG)		MLEE corpus; PubMed	250 abstracts; 350 abstracts	18 types	40 types	[47]
	Pathway curation (PC)		PubMed	525 abstracts	4 types	23 types	[48]
	Gene regulation ontology (GRO)		MEDLINE	300 abstracts	174 types	126 types	[49]
	Gene regulation network in bacteria (GRN)		PubMed	201 sentences	6 types	12 types	[50]
DDIExtraction 2013 challenge (SemEval-2013 Task 9)	DDI corpus	2013	DrugBank; MEDLINE	6795 sentences/792 texts; 2147 sentences/233 abstracts	4 pharmacological mentions	Mechanism, effect, advice, int	[19]
BioCreative V CDR task	BC5CDR corpus	2016	PubMed	1500 abstracts (500 for training, 500 for development, 500 for test)	Chemical/disease	Binary interaction	[36]
BioCreative VI ChemProt task	ChemProt corpus	2017	PubMed	2432 abstracts (1020 for training, 612 for development, 800 for test)	Chemical compound/drug, gene/protein	22 types	[37]
BioCreative VI PrecMed task	Precision medicine (PM)	2017	PubMed	5509 abstracts	Protein	Binary interaction	[38]
MADE 1.0 challenge	Medication and adverse drug event from electronic health records (MADE1.0) corpus	2018	University of Massachusetts Memorial Hospital	1089 EHR	9 types	7 types	[54]
2018 n2c2 shared task-track 2	2018 n2c2 track 2 dataset	2018	MIMIC-III	505 discharged summaries (303 for training, 202 for test)	9 types (drugs and 8 other types)	8 types (drugs with 7 other types)	[52]
BioCreative VII DrugProt shared task	DrugProt corpus	2021	PubMed	5000 abstracts (3500 for training, 750 for development, 750 for test)	Chemical compounds (drug included), gene/protein	13 types	[40]

**Table 2:** Overview of relation extraction datasets

Dataset name	Published	Data source	Scale/data size	Relation objects	Relation types	Reference
IEPA	2002	PubMed	486 sentences/ ~300 abstracts	Chemicals	Binary interaction	[13]
AIMed	2005	MEDLINE	1955 sentences/ 225 abstracts	Human protein/gene	Binary interaction	[15]
LLL	2005	MEDLINE	77 sentences	Protein/gene in *Bacillus subtilis*	3 types	[14]
Bioinfer	2007	PubMed	1100 sentences	Protein/gene/RNA and related	68 types	[16]
HPRD50	2007	MEDLINE	145 sentences/ 50 abstracts	Human protein/gene	Binary interaction	[17]
EMU	2010	PubMed	109 abstracts on mutation	Human protein/gene, disease (prostate cancer/breast cancer)	Binary interaction	[55]
MLEE corpus	2012	PubMed	2608 sentences/ 262 abstracts on angiogenesis	Organism, anatomy, molecule types (14 entity types included in 3 major entity types)	Anatomical, molecular, general, planned events (18 events included in 4 major types)	[56]
EU-ADR	2012	MEDLINE	300 abstracts	Drug, disease, and target (gene, protein and sequence variation)	drug–disease, drug–target, target–disease	[57]
ADE corpus	2012	MEDLINE	20 967 sentences/ 2972 documents	Drug, adverse effect and dosage	Drug–adverse effect, drug–dosage	[21]
GAD corpus	2015	PubMed	5329 sentences	Gene and disease	Binary interaction	[58]
PhenoCHF	2015	i2b2 recognizing obesity challenge; PMC	300 discharged summaries; 10 full-texts	Six COPD-related mentions	3 types	[59]
BRONCO	2016	PMC	108 full-texts	Variant, gene, disease, drug and cell-line	Variant with other entities (4 types)	[60]
N-ary	2017	PMC	264 867 sentences	Drug, gene, mutation	Six types (5 pos, 1 neg)	[61]
DDAE dataset	2019	PubMed	521 abstracts (400 for training, 121 for test)	Disease	2 types	[62]
RENET2	2021	MEDLINE; PMC	1000 abstracts; 500 full-texts	Gene and disease	Associated, non-associated and ambiguous	[63]

BioCreative has been a well-established text-mining community in biology since 2004. One task from the BioCreative II competition in 2006 [34] used 1098 full-text biomedical articles from PubMed as the main source of information. These articles were compiled for the interaction pair subtask (IPS) after annotation by domain experts. In 2009, the BioCreative II.5 interaction pair task (IPT) dataset was sourced from FEBS Letters articles, with only 122 full-texts containing PPI annotations [35]. In 2016, BioCreative V introduced a task to capture chemical–disease relationships (CDRs) [36], and BioCreative VI featured a task to study chemical–protein interactions [37]. Both datasets were collected from PubMed abstracts, with the BioCreative V BC5CDR corpus comprising 1500 abstracts and the BioCreative VI ChemProt corpus containing 2432 abstracts. BioCreative VI PM [38] includes 5509 PubMed abstracts from IntAct/Mint [39]. PPI relations are annotated with those interacting protein pairs if the mutations affect the interactions. BioCreative has expanded the scope of its tasks to include a variety of biomedical relations, ranging from general protein–protein interactions (PPIs) to more specific chemical–disease interactions. In 2021, BioCreative VII introduced a track focused on drug and chemical–protein interactions (DrugProt) [40], using 5000 PubMed abstracts with mentions of genes and chemical compounds. This task is designed to promote the development and evaluation of systems for detecting relations between chemical compounds/drugs and genes/proteins.

Another important text-mining competition in the biomedical field is the BioNLP-ST. BioNLP-ST has held the Genia event (GE) task in RE since 2009 [41] and repeated it in BioNLP-ST 2011 [42]. The 2011 abstract collection uses the same data as the 2009, which originates from the GENIA corpus [43] by Kim, to measure the progress of the scientific community. Fourteen full-text papers are annotated to evaluate the applicability of the text. Three additional RE tasks were published in the same year, namely the entity relations (REL) task, the infectious diseases (ID) task and the epigenetics and post-translational modifications (EPI) task [44]. The REL task focuses on supporting the main event extraction task by independently identifying entity relations. The ID task deals with the molecular mechanisms of infectious diseases, which include various types of molecular entities, disease-causing microorganisms and other organisms affected by the diseases. The goal of EPI task is to extract events related to chemical modifications of DNA and proteins, particularly those related to the epigenetic control of gene expression. In 2013 [45], five tasks were included in the competition: GE extraction, cancer genetics (CG), pathway curation (PC), gene regulation ontology (GRO) and gene regulation network in bacteria (GRN). These tasks involve relationships ranging from 12 to 126 types, depending on the complexity of the topic [46–50].

The 2010 i2b2/VA challenge (now termed n2c2) focuses on the relations between treatments and tests, using 871 EMRs from three medical institutions [51]. The 2018 n2c2 shared task-track 2 [52] uses the Medical Information Mart for Intensive Care-III (MIMIC-III) clinical care database [53] to extract medication information from 505 discharge summaries. These challenges have relation classifications based on the drug and its related information, but identifying certain relation types, such as reason-drug, can be quite error-prone due to hidden evidence and confusing information in adverse drug events (ADEs).

Other competitions include the DDIExtraction, which holds challenges focused on the identification of DDI in 2011 and 2013 [18–20], using DrugBank and MEDLINE as sources of target literature. These challenges, respectively, have 579 and 792 texts. In 2018, the MADE 1.0 challenge uses 1089 hospital EHRs to discuss medication and ADEs [54]. This challenge defined seven relation types among nine NE types and featured four related relations of Drugname–Dosage, Drugname–Route, Drugname–Frequency and Drugname–Duration. The latter two challenges, which may present cross-sentence relations, are difficult to extract. For more details, Table 1 summarizes these RE datasets and their challenges.

Several corpora for event extraction have been released in recent decades. Doughty *et al.* [55] developed a technique that quickly scans PubMed abstracts to find mutations associated with prostate (PCa) and breast cancer (BCa). After analyzing, they identified 51 mutations related to PCa and 128 mutations related to BCa from 109 abstracts. Table 2 lists many papers releasing RE datasets without challenges. Pyysalo *et al.* [56] presented the multi-level event extraction (MLEE) corpus, which has ontological foundations and annotates target types and entities as events. The MLEE corpus is comprised of 262 abstracts collected from PubMed and partially adopted for the CG task in BioNLP-ST 2013. Another corpus, the EU-ADR, was published in 2012 and focuses on extracting information about drug–disease, drug–target and target–disease relationships [57]. It contains 300 abstracts annotated by domain experts from MEDLINE. Both entity-based and relation-based annotations achieve an average of 76.2–77.6% good inter-annotator agreement (IAA). The ADE corpus, which focuses on extracting information about drug-related adverse effects from medical case reports, contains nearly 30 000 documents from MEDLINE and randomly selected 3000 for annotation and benchmarking. Bravo *et al.* [58] developed a new gene-disease association corpus, the GAD corpus, using a semi-automatic annotation procedure. The corpus includes 5329 relations, and each relation is expressed in one sentence from PubMed. The PhenoCHF corpus concerns phenotype-disease associations in discharge summaries from 300 congestive heart failure (CHF) patients and is annotated with three types of information: cause, risk factors and sign and symptom [59]. It aims to support the development of text mining systems that can obtain comprehensive phenotypic information from multiple sources. Another corpus, the Biomedical entity Relation ONcology COrpus (BRONCO), contains more than 400 variants and their relationships with genes, diseases, drugs and cell lines, as documented in 108 PMC full-text articles [60]. BRONCO specifically collects papers published in cancer research due to the high occurrence of mutation mentions in that field. What is special is that, even though *N*-ary [61] focuses mainly on 59 different drug–gene–mutation triples from the knowledge base rather than pairs, they still extend the relations between drug–genes and drug–mutations, resulting in 137 469 drug–gene and 3192 drug–mutation positive relations. The DDAE corpus, specifically discusses the relationship between comorbidities (disease–disease association, DDA) [62]. It covers 521 abstracts from PubMed and defines positive (correlated), negative and null relations to determine the link between two disease entities. In RENET2 [63], it proposed a model and dataset to extract gene-disease associations. They reannotate the previously annotated 500 abstracts (RENET [64]) and use three gene-disease pairs to automatically annotate another 500 abstracts. Finally, they annotate 500 unlabeled full-text PMC articles using the model trained on 1000 abstracts. Finally, there are five regular PPI benchmark datasets available for information extraction development: AIMed [15], BioInfer [16], HPRD50 [17], IEPA [13] and LLL [14], which are listed in Table 2. Comparisons among the five datasets demonstrate the variability of PPI [65]. AIMed and BioInfer contain over 1000 sentences and include all occurring entities, while HPRD50, IEPA and LLL have smaller datasets and limit entity scopes to particular terms. Therefore, AIMed and BioInfer generally demonstrate lower performance in machine learning systems. Pyysalo *et al.*’s [65] experimental results showed that the average difference in *F*-measure between the PPI corpora is 19%, with even wider differences in some cases. This may be due to the diversity of PPI mentions across the datasets.

### Overview of RE systems

In the field of text mining for biomedical RE, strategies have evolved over the years, falling into four categories: rule-based, traditional ML-based, traditional deep learning (DL)-based and transformer-based methods. These approaches have transitioned from rule-based systems to transformer models. Subsequently, we will provide examples of methods within each category and their respective performance on RE datasets.

#### Rule-based

Rule-based methods utilize a pre-defined word list and annotated rules to find relations [66, 67] and use patterns [68, 69] composed of regular expressions or filtered through parsing and tagging structures. RelEx [17] is a rule-based RE system, which combines dependency parsing trees, part-of-speech (POS) tagging and noun-phrase-chunking for better accuracy. RelEx achieves an *F*-measure of 44% on the AIMed dataset [65]. Yakushiji *et al.* [70] used predicate-argument structures (PASs) for automatic pattern construction to produce generalized patterns compared with surface structures of words. It achieves an *F*-measure of 33.4% on AIMed. Manual rule construction by domain experts is time-consuming and labor-intensive, so some studies have proposed automatically learning patterns [15, 71] as a solution. RAPIER [72] used a pattern learning algorithm that incorporates several inductive logic programming systems and acquires unbounded patterns for extracting information from texts. However, RAPIER achieves an *F*-measure of 21.0% on AIMed [15], while the dictionary concatenated with the generalized RAPIER system obtains an *F*-score of 52.81%. Some studies collect potential trigger evidence to predict relation occurrence. Huang *et al.* [71] mine verbs that describe protein interactions. PKDE4J [73] constructs a bio-verb dictionary derived from Sun *et al.* [74] to investigate relation types. PKDE4J reaches an *F*-measure of 47.0% on the CAD corpus and 83.8% with rules, such as nominalization, negation, containing a clause and entity counts. However, rule-based models can be difficult to adapt to new datasets.

#### Traditional ML-based

ML-based approaches can be used when a large-scale manually annotated corpus is available. RE can be formulated as classification problems, where entities are represented as vectors or objects. These techniques use detected features or patterns to classify sentences containing relations, similar to statistical approaches based on words frequently co-occurring in a context. Support vector machine (SVM) is a traditional statistic classification method [75] used in RE tasks for its effectiveness in text classification [76]. With POS tags, the output of the dictionary-based protein tagger, suffix features and other settings, the SVM reaches an *F*-measure of 54.42% on AIMed [15]. Kernel-based methods, such as SVM-based or other ML methods, can be applied alone or in combination in RE tasks [77–81], and they have proven to be effective.

#### Traditional DL-based

DL techniques, specifically neural networks (NNs), have been highly effective in RE tasks. When a NN learns representations from multiple hidden layers, it is referred to as a deep NN (DNN). This learning method is referred to as DL [82]. In recent years, DNN systems, such as convolutional NNs (CNNs) and recurrent NNs (RNNs), have been efficient at encoding the semantic features of entities and sentences in RE tasks [83]. CNNs have been known to consistently extract the most valuable features in a flat structure. The CNN model achieves an *F*-measure of 69.75% on the DDI corpus [84]. By using CNNs and MaxEnt models for RE at inter- and intra-sentence levels separately, an *F*-measure of 61.3% is reached on the BC5CDR corpus [85]. Peng *et al.* [86] proposed a multi-channel dependency-based CNN (McDepCNN) model, which earns an *F*-measure of 63.5% and 65.3% on AIMed and BioInfer, respectively. RNNs have the advantage of being able to learn from long word sequences. Hsieh *et al.* [87] proposed a bi-directional (Bi) RNN model with two long short-term memory (LSTM) components, where the hidden layer is concatenated with the forward and backward output vectors. Their best Bi-LSTM system achieves an *F*-measure of 76.9% and 87.2% on AIMed and BioInfer, respectively, without any feature engineering. Using shortest dependency path (SDP) representations between two entities as input for the Bi-LSTM model, an *F*-measure of 71.4% is obtained on the ADE corpus [88]. Lim *et al.* [89] proposed a tree-LSTM model with another RNN model, stack-augmented parser interpreter NN (SPINN), which obtains an *F*-measure of 64.1% on the ChemProt corpus. DL-based systems are increasingly hybridizing two or more NN models to improve performance [90].

#### Transformer-based

Recently, transformer-based models, such as Bidirectional Encoder Representations from Transformers (BERT) [91], have been shown to be effective in RE. BERT represents a robust language model that is jointly conditioned on both left and right contexts in all layers. The pre-training corpora are BooksCorpus (800 million words) and English Wikipedia (2500 million words). BERT can be further fine-tuned for specific tasks and has been shown to improve performance in general domains, such as GLUE. Following this trend, bio-medical BERT (BioBERT) [92] is proposed, which is derived from BERT with a pre-trained model based on biomedical literature. In BioBERT v1.1, it achieves an *F*-measure of 79.83%, 79.74% and 76.46% on GAD corpus, EU-ADR and ChemProt corpus, respectively. When applied to PPI tasks, BioBERT achieves an *F*-measure of 66.7% and 67.7% on AIMed and BioInfer, respectively [93]. BioBERT adopts the attention mechanism on the last output layer [94], which improved the *F*-measure by 0.34% on the ChemProt corpus compared with the prior results. Through revisions to its architecture, it achieves an even better *F*-measure of 82.5% and 80.7% on the PPI corpus and DDI corpus, respectively. Another BERT variant, Blue-BERT [95], improves an *F*-measure of 3.52–63.61% on the BC5CDR corpus, where the pre-trained data include MIMIC-III clinical notes. BERT-GT [96] is a novel model that adds a graph transformer (GT) architecture to BERT and achieves an *F*-measure of 65.99% on the BC5CDR corpus. Other biomedical pre-trained language models (BioPLMs) also perform well on BioNLP tasks [97]. Besides BioBERT and BlueBERT, other three BioPLMs are used on the PEDD dataset, and the results are described in the Challenge Results session. New studies have developed hybrid approaches by combining various RE techniques for better performance.

### Compilation of the PEDD

The PEDD dataset provides the entity pair and target relation type during the training stage, while potential relation evidence, such as trigger words, is embedded within the texts. The goal for machine models is to effectively extract valuable information and correctly classify the targets into the appropriate classes. In the subsequent section, we will clearly outline the process of compiling the PEDD dataset, including data collection, annotation and statistics.

### Data collection

**Figure 2:**
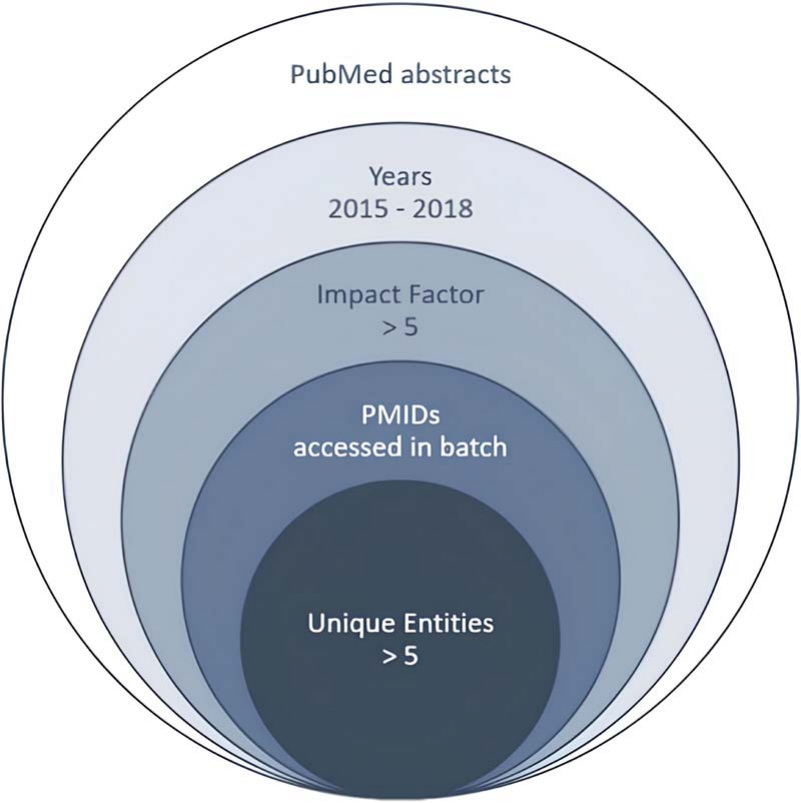
Flowchart of the PEDD data collection process.

The PEDD dataset was collected from PubMed, and several conditions were applied to retrieve the ideal documents. The focus was on studies published from 2015 to 2018, as they represented the latest biomedical research information at that time. Only abstracts with impact factors >5 were recruited to maintain good scientific quality. Instead of querying specific topics or keywords, articles in the PEDD were accessed in batches by PMID. After filtering the articles with the above-mentioned specifications, abstracts with more than five unique protein entities were used as the final target texts, thereby guaranteeing the occurrence of a potential relation. A flowchart illustrating the data collection process is given in Figure 2.

Considering the aforementioned data collection process, several critical issues require in-depth discussions. The compilation of the PEDD dataset focuses on single-sentence relation extraction, while cross-sentence relation extraction presents a more complex challenge. This advanced task demands dealing with context understanding, ambiguities, pronouns and coreference resolution, elevating the burden for annotation training to build an ideal corpus of a similar data scale to the PEDD dataset. Furthermore, few-shot learning poses another intricate issue in handling limited data, with the risk of model overfitting. To minimize this phenomenon in the PEDD dataset, we only included articles with five or more independent NEs as annotation targets. This approach, without deliberately excluding abstracts without PPI, increases the likelihood of relationship occurrences, achieving a relatively balanced ratio of positive to negative data. This provides sufficient samples and reduces the limitations of few-shot learning. We further discuss these challenges in the ‘Conclusion’ section to highlight the complexities of the RE topic.

### Data annotation

The PPIE competition was created to advance the development of biomedical RE systems. The PEDD dataset was annotated by three experts, including a Biomedical Informatics Ph.D. leader and two annotators with master’s degrees in molecular biology and biomedicine.

Given the wide range of interactions between proteins, we have identified several relevant interaction types of value to biologists. Additionally, we clearly define the scope of the ‘Protein’ entity before starting the annotation process. By incorporating these two crucial elements, the annotation guidelines become more easily understood.

#### Definition of the ‘protein’ entity

To facilitate protein entity identification in all abstracts prior to relation identification, we utilized the GENE bioconcept annotations from Pubtator [36] for pre-labeling. These annotations encompass various gene-related entities, including proteins, DNA and miRNA. Considering the significant impact of miRNA on signal transduction and protein biosynthesis [98, 99], we have expanded the definition of ‘protein’ entities to accommodate the entity pre-annotations with distinct Entrez IDs [100]. It is important to note that the BioNLP-ST2011/2013 GE task datasets in Table 1, as well as the AImed and Bioinfer datasets in Table 2, also include gene-related entities in addition to protein-type entities. Furthermore, this expansion aims to minimize the effort required to distinguish entity properties. It’s worth noting that earlier PPI datasets, such as Aimed and Bioinfor, did not include miRNA within their entity scope, possibly due to the limited attention given to miRNA-related issues during that time [101].

**Figure 3:**
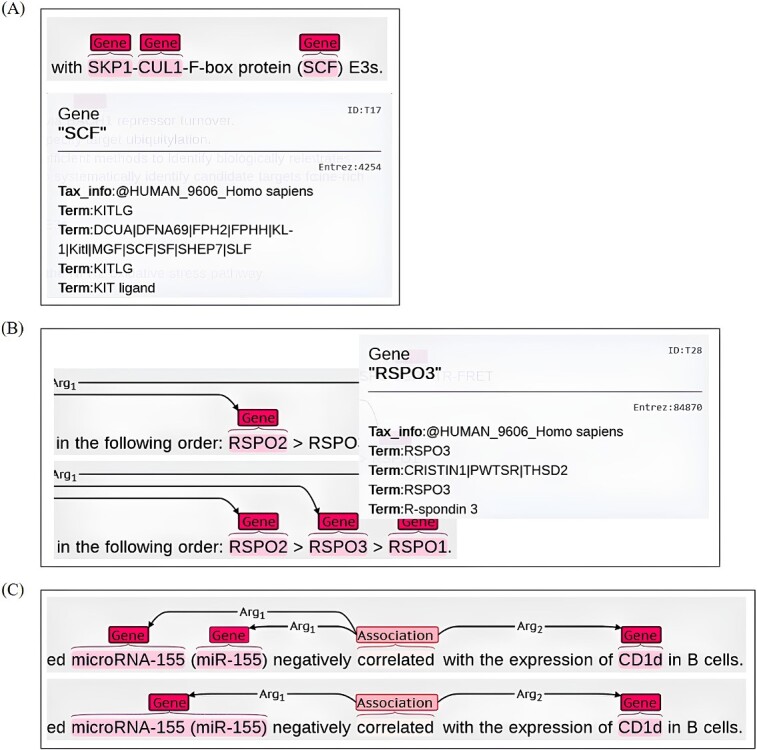
Three annotation scenarios (A-C) for pre-labeling revision. (A) the instance of incorrect pre-labeling gene entity (B) the missed gene entity 'RSPO3' within a relation is added to ensure information integrity (C) merge the two miRNA entities into one for removing redundant relations.

Before confirming a relation, we occasionally make modifications to the Pubtator labels to ensure the accuracy of the content in the following three scenarios:

(i) In cases where an entity is linked to an incorrect Entrez ID, we remove the original tag from Pubtator. For instance, in Figure 3A, the mention of ‘*SCF*’ is erroneously tagged as a distinct gene entity. However, ‘*SCF*’ actually represents the abbreviation of the ‘*SKP1-CUL1-F-box*’ protein complex. By removing such similar cases, we aim to reduce noise and improve the accuracy of the annotations.
(ii) In certain cases, we address the omission of a protein entity by adding a new entry with the corresponding Entrez ID when it is found to have a relationship with another entity. This is exemplified in Figure 3B, which illustrates corresponding scenarios. In the given instance, all RSPO1–3 proteins exhibit a specific relationship with other entities, but ‘*RSPO3*’ was inadvertently overlooked during the initial pre-labeling process. To rectify this oversight and ensure the inclusion of potential relations, our annotators diligently revise and update the annotations accordingly.
(iii) To avoid generating redundant relations, we merge neighboring entities that share the same ID. This practice is exemplified in Figure 3C, which accurately represents this phenomenon. The default annotation of Pubtator assigns separate labels to the full name and acronym of ‘*microRNA-155*’, resulting in repetitive relations with the ‘*CD1d*’. In order to achieve optimal presentations, we consolidate the microRNAs into a single entity, thereby eliminating the redundancy caused by independent labeling of different entity representations.

#### P‌PI relation types

In previous datasets, the PPI relationships were predominantly presented as binary classifications. However, there was a lack of deeper exploration into the intricate connections associated with protein regulation, translational modification and signaling transduction. Domain experts conducted data inspection using a random sample of 1500 biomedical abstracts to reach a consensus on defining PPI relations with greater refinement. This refined definition will contribute to valuable further studies in the field.

**Figure 4:**
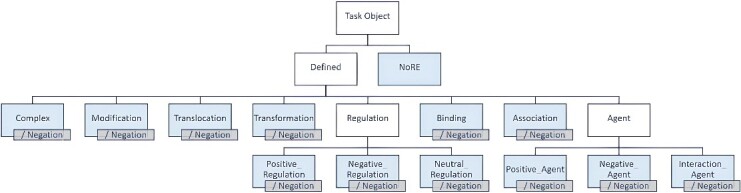
PPI relation types. All relation types additionally have a Negation attribute when content contraindicates the relation’s occurrence. There are a total of 24 types of PPI, excepting NoRE.

Based on the data observations, PPI relations have been categorized into 12 categories, as depicted in Figure 4. These categories include ‘Complex’, ‘Modification’, ‘Translocation’, ‘Transformation’, ‘Regulation’, ‘Binding’, ‘Association’ and ‘Agent’. The ‘Regulation’ category is further divided into ‘Positive_Regulation’, ‘Negative_Regulation’ and ‘Neutral_Regulation’, while the ‘Agent’ category is subdivided into ‘Positive_Agent’, ‘Negative_Agent’ and ‘Interaction_Agent’. All relation types include the ‘Negation’ attribute when the description denies the occurrence of the relation. Additionally, entity pairs can exhibit multiple relations within the same sentence. We provide further definitions and examples for each relation type in the subsequent sections.

##### Complex

When two or more concrete proteins form a complex in the same statement with words, such as ‘complex’, ‘dimer’, ‘trimer’, ‘sliding clamp’, etc., they are considered to have a Complex relation. However, a chimeric protein is not considered a complex, as the chimeric form represents neighboring proteins that are joined in sequence rather than forming a complex. Furthermore, when a complex name is tagged as an entity, the term is not considered to be in a Complex relation with other potential complex subunits.

Instance:

*‘Double-strand RNA promoted RALB ubiquitylation and SEC5-TBK1 complex formation.’*

*Excerpted from PMID 24056301.*

Negative Instance:

*‘Drosophila Atg17 is a member of the Atg1 complex as in mammals, ….’*

*Excerpted from PMID 24419107.*

##### Modification

Modification refers to the occurrence of post-translational modifications (PTM), such as phosphorylation, methylation and ubiquitination in proteins. This category implies that one protein is modifying another through a certain enzymatic or chemical process.

Instance:

*‘Recombinant NEDD4-1 promoted Mdm2 ubiquitination in vitro in a concentration- and time-dependent manner.’*

*Excerpted from PMID 24413081.*

##### Translocation

When a protein entity causes the movement of its interactor within the same sentence, the entity pair is identified as being related through translocation. Various phrases may serve as evidence of this relation, such as localize, recruit, internalization, nuclear accumulation and other similar terms. It indicates that one protein is moving another protein from one location to another in the statement.

Instance:

*‘The I377M mutation and Fbxo4 deficiency result in nuclear accumulation of cyclin D1, a key transforming neoplastic event.’*

*Excerpted from PMID 24019069.*

*‘Tat favors the secretion of interleukin-2, interferon-γ and granzyme B in CD8+ T cells.’*

*Excerpted from PMID 24841128.*

##### Transformation

When a sentence describes a change in protein structure related to a protein entity pair, the relation is identified as transformation. Certain phrases can help clarify this relationship, such as dimerize, polymerize, assemble, disassemble, dissociate and other similar terms. It indicates that one protein is changing the structure of another protein.

Instance:

*‘In vitro studies show that MCM-BP disassembles MCM2-7 bound to DNA with a fork-like structure by interacting with MCM3, MCM5, and MCM7.’*

*Excerpted from PMID 24299456.*

##### Regulation

Regulation relation types are annotated when the expression level or activity of a target entity is regulated or altered by another. The relation mentioned is completed by attaching appropriate modifiers to the context, such as ‘positive’, ‘negative’ or ‘neutral’. The context is important in order to determine the regulation relation; different expressions might indicate distinct regulation relations.

##### Positive_Regulation

This regulation type is applied when the expression level or enzymatic activity of a protein entity is increased by another entity. Words, such as induce, stimulate, upregulate, augment, activate and reestablish, can serve as evidence for this relation. It indicates that one protein is promoting or increasing the activity or expression level of another protein.

Instance:

*‘HIV-1 Tat is known to up-regulate CCL5 expression in mouse astrocyte, but the mechanism of upregulation is not known.’*

*Excerpted from PMID 24299456.*

##### Negative_Regulation

In contrast, Negative_Regulation is applied when the expression level or enzymatic activity of a protein entity is decreased by another entity. Words, such as decrease, downregulate, inactivate, interfere, suppress and other similar terms, can indicate this relation. It indicates that one protein is decreasing or inhibiting the activity or expression level of another protein.

Instance:

*‘Mast cell chymase degrades the alarmins heat shock protein 70, biglycan, HMGB1, and interleukin-33 (IL-33) and limits danger-induced inflammation.’*

*Excerpted from PMID 24299456.*

##### Neutral_Regulation

The neutral relation is appropriate when it is difficult to discern the expression profiles of a target entity. Words, such as mediate, drive, modulate, affect, control, influence, desensitization and other similar terms, can indicate this relation. They indicate that one protein is affecting the activity or expression level of another protein in a way that is not clear as positive or negative.

Instance:

*‘Furthermore, GAREM2 and Shp2 regulate Erk activity in EGF-stimulated cells.’*

*Excerpted from PMID 24003223.*

##### Binding

Physical interactions that are not correlated with the relations mentioned above are tagged as Binding relations. Words, such as bind, target, recognize, occupy, harbor and hijack, can be critical in establishing this relation. They indicate that one protein is physically interacting with another protein, but not in a way that can be classified as other relations, such as modification, regulation, etc.

Instance:

*‘Gtr1p and Gtr2p bind Ego1p and Ego3p, which are tethered to the endosomal and vascular…’*

*Excerpted from PMID 24702707.*

##### Association

The Association relation denotes that the given PPI is vague or indirect. Words or phrases that indicate weak linkages include dependent, association, interaction, require, colocalize, in response to and cooperate. This relation type is less specific than others, indicating that the two proteins exhibit a certain interaction, but it is not specified how or if it is a weak interaction or not. For example, in the given context, genes, such as *hus1*, *gadd45a*, *rb1*, *cdkn2a* and *mre11a*, all present an Association relation with *per2*. This might indicate that these genes are interacting with or dependent in some way to *per2*, but the nature of this interaction is not specified.

Instance:

*‘Expression of cell cycle regulatory factors hus1, gadd45a, rb1, cdkn2a and mre11a correlates with expression of clock gene per2 in human colorectal carcinoma tissue.’*

*Excerpted from PMID 24062075.*

##### Agent

The Agent relation is applied to a target entity that serves as an executor for its interaction object. This relation can be divided into three subtypes similar to Regulation. Phrases, such as via, by and through, can also serve as evidence of Interaction_Agent when two entities in the text content link to each other in this manner. It indicates that one protein is taking some action on another protein but does not specify the nature of that action.

##### Positive_Agent

This relation type applies when a positive executor, such as an activator or inducer, serves as evidence of linkage between two entities.

Instance:

*‘Thus, not only is c-FLIP the initiator of caspase-8 activity during T cell activation, cell growth.’*

*Excerpted from PMID 24275659.*

##### Negative_Agent

This relation type applies when a negative executor, such as an inhibitor or suppressor, serves as evidence of linkage between two entities.

Instance:

*‘Mdm2 is a critical negative regulator of the tumor suppressor protein p53.’*

*Excerpted from PMID 24413081.*

##### Interaction_Agent

This relation type applies when a neutral executor, such as an upstream or downstream component, or an instrumental preposition (e.g. via and by) is used as evidence of linkage between two entities.

Instance:

*‘We show that MDM2 is an E3 ligase for K48-linled EID1 ubiquitination for its degradation.’*

*Excerpted from PMID 24167073.*

##### Negation

All of the aforementioned relation types can have the Negation attribute added when the text contradicts the occurrence of the relation. In this example sentence, *Gpr22* presents a negation of an Interaction_Agent relation with *Foxj1a* and *Rfx2*.

Instance:

*‘Further, we found that Gpr22 does not act upstream of the two cilia master regulators, Foxj1a and Rfx2.’*

*Excerpted from PMID 25335082.*

#### The major taxonomy of PPI relation types

To simplify the classification of PPI relation types, six major categories can be used to encompass all the aforementioned relation classes. These are ‘Causal_Interaction’, ‘General_Interaction’ and ‘Regulation’ as well as their corresponding negation categories.

(i) ‘Causal_Interaction’ includes PPIs that exhibit a cause-effect relationship, such as ‘Modification’, ‘Translocation’ and ‘Transformation’.
(ii) ‘General_Interaction’ includes PPIs that do not present a clear causal relationship between the interacting components, such as ‘Association’, ‘Binding’, ‘Complex’ and ‘Agent’.
(iii) ‘Regulation’ includes PPIs that relate to the expression level or protein activity regulation, such as ‘Positive_Regulation’, ‘Negative_Regulation’ and ‘Neutral_Regulation’.

### The scope of irrelevant PPI

While the PPI relation types discussed earlier consider various interaction features, some PPI-like relations are excluded due to patterns that do not meet the definition of PPI. The distinct criteria outlined below highlight the principles used to eliminate relations, along with corresponding examples of non-relation cases.

(i) Self-relation is not considered, even though auto-regulation is common in natural phenomena. Therefore, interactions involving a single Entrez ID are removed.

Negative Instance:

*‘Prevention of TPP1 ubiquitination prolonged TPP1 half-life ~2-fold from 45 min …’.*

*Excerpted from PMID 25172512*

(ii) Protein interactions with a gene family, pathway, axis, cell, disease, population, ortholog, homolog, paralog, biochemical process or physiological process are not considered as PPI relations.

Negative Instance:

*‘Tmc2a is an ortholog of mammalian TMC2, which along with TMC1 has been implicated in mechanotransduction in mammalian hair cells.’*

*Excerpted from PMID 25114259*

(iii) Speculative results, hypotheses and unspecific statements are excluded. Words, such as may, might, should, possible, perhaps and could be, are used to identify speculative sentences.

Negative Instance:

*‘The results indicate a significant role for the AKAP5 scaffold in signaling and trafficking of the* *β* *1-AR in cardiac myocytes and mammalian cells.’*

*Excerpted from PMID 24121510.*

*‘Nedd4-2 regulates surface expression and may affect N-glycosylation of hyperpolarization-activated cyclic nucleotide-gated (HCN)-1 channels.’*

*Excerpted from PMID 24451387*

(iv) In some situations, major relations rely on the formation of sub-relations, such as Complex and Association. If the sub-relations fail to be established, the dependent relation would not be established. For example, if AKAP5 forms a complex with PKA, and the complex subsequently targets β1-AR, but PKA is not referred to as a protein with a discrete Entrez ID because it is composed of several unique protein subunits, the complex relation between AKAP5 and PKA is omitted, and the following potential relation with β1-AR is interrupted.

Negative Instance:

*‘Furthermore, recycling of the* *β* *1-AR in rat neonatal cardiac myocytes was dependent on the targeting the AKAP5-PKA complex to the C-terminal tail of the* *β* *1-AR.’*

*Excerpted from PMID 24121510.*

### IAA analysis

To assess IAA in the PEDD, we employed Cohen’s kappa coefficient [102] to measure the consistency of annotation [103]. The kappa value (*k*) is calculated using Equation (1), where ${P}_0$ represents the observed agreement between annotators and ${P}_e$ is the hypothetical probability of chance agreement


(1) \begin{equation*} k=\frac{P_0-{P}_e}{1-{P}_e}. \end{equation*}

The kappa value ranges from −1 to 1, where value of 1 indicates perfect agreement and value of 0 indicates agreement no better than expected by chance [104].

The PEDD corpus was annotated by a team of three annotators. The IAAs for binary relations (Level 1) and relation types (Level 2) were evaluated and found to be consistently >0.8 on average, as presented in Table 3. This suggests a high degree of agreement among annotators, indicating that the annotations in the PEDD dataset are reliable and consistent. According to Altman’s interpretation of kappa values, the PEDD annotation achieved almost perfect agreement [104], which provides a strong foundation for further research in this area.

**Table 3:** IAA scores of the PEDD dataset

	L1 (binary relation)	L2 (relation types)
Annotator A-B	0.890	0.86
Annotator B-C	0.911	0.89
Annotator C-A	0.899	0.863
Average value	0.9	0.871

## COMPETITION DETAILS

The distribution of PPI relation types in the divided dataset is shown in Table 4, which includes 23 types of relations. However, the annotation guideline excludes two minor categories: Negative_Negative_Agent and Negative_Positive_Agent. To facilitate initial model building, the PPIE track datasets are divided into smaller sections and released incrementally. For the PEDD dataset, the following steps were taken:

(i) Step 1: A sample set of 150 documents was released.
(ii) Step 2: Train1 (1400 documents) and Dev1 (500 documents) were released, and participants could upload and evaluate predictions for Dev1.
(iii) Step 3: Train2 (2700 documents) and Dev2 (500 documents) were released.
(iv) Step 4: A test set of around 13 600 documents was released, but only 1500 texts were annotated for scoring. During the final stage, the remaining portions of the test set were provided without manual annotations. Evaluation was performed only on the specific annotated sets, not the entire test set submitted by participants. The top 1–3 system predictions were considered for ranking based on these evaluations. After the upload deadline passed, the private leaderboard was revealed, and the competition was ranked based on major parts of the annotated data.

**Table 4:** PPI relation types for each dataset in PEDD. The datasets include sample set, training set part one and part two (Train1 and Train2), development set part one and part two (Dev1 and Dev2), and test set

RE_Type	Sample	Train1	Train2	Dev1	Dev2	Test	Total
Association	299	1014	1963	392	387	889	4944
Binding	86	354	631	141	107	347	1666
Complex	181	365	745	170	160	320	1941
Interaction_Agent	103	552	1144	180	214	496	2689
Modification	79	296	563	98	145	288	1469
Negation_Association	19	30	56	11	13	22	151
Negation_Binding	3	7	16	4	-	7	37
Negation_Interaction_Agent	2	3	3	4	4	2	18
Negation_Modification	7	12	23	6	10	13	71
Negation_Negative_Regulation	3	9	37	2	2	11	64
Negation_Neutral_Regulation	3	18	28	2	9	16	76
Negation_Positive_Regulation	3	15	27	12	10	18	85
Negation_Transformation	-	10	10	2	4	5	31
Negation_Translocation	1	2	7	5	-	14	29
Negative_Agent	8	39	74	17	23	38	199
Negative_Regulation	103	667	1614	282	367	736	3769
Neutral_Regulation	73	459	968	177	215	569	2461
NoRE	2304	22 282	69 948	9075	16 254	37 353	157 216
Positive_Agent	3	29	46	10	6	17	111
Positive_Regulation	111	837	1994	352	472	1010	4776
Transformation	13	48	73	15	14	54	217
Translocation	51	174	360	67	44	220	916
Negation_Complex	1	–	–	–	–	–	1
Total	3456	27 222	80 330	11 024	18 460	42 445	182 937

Table 5 provides statistics for all datasets. In total, PEDD contains 6823 documents containing 182 937 PPI relations involving 18 874 unique genes. On average, each document contains 5.7 sentences, and 26.8 gene pairs have PPI relations. Compared with other similar PPIE datasets, PEDD has a larger number of documents, which can enhance the capability of trained machines. Table 6 shows an example of the tab-delimited format for training data, which includes the PMID from the original PubMed article, a sentence from the article, a sentence ID, gene names with Entrez Gene IDs, the start/end indexes of the gene pair and the PPI relation type to be predicted. Note that only the sample and training sets provide PPI relation types. Multiple gene pairs are often involved in one sentence, making it necessary for participants to overcome such obstacles and retrieve the exact interaction events.

**Table 5:** Statistics for each sub-dataset in PEDD

Dataset	Documents	Sentences	Gene pairs	Unique genes
Sample	149	861	3456	930
Train1	1400	7714	27 222	3548
Train2	2773	16 273	80 330	6563
Dev1	500	2814	11 024	1682
Dev2	500	3195	18 460	2134
Test	1501	8631	42 445	4017
Total	6823	39 488	182 937	18 874

**Table 6:** Data features in PEDD

PMID	Sentence_ID	Sentence	Gene1|Gene1_ID	Gene1_Index(start|end)	Gene2|Gene2_ID	Gene2_Index(start|end)	RE_Type
24702707	S1	Gtr1p and Gtr2p bind Ego1p and Ego3p, which are tethered to the endosomal and vascular…	Gtr1p|854918	0|5	Ego1p|853876	21|26	Binding
24702707	S1	Gtr1p and Gtr2p bind Ego1p and Ego3p, which are tethered to the endosomal and vascular…	Gtr2p|853072	10|15	Ego1p|853876	21|26	Binding

### Evaluation metric

In the PPIE track, we evaluate the performance of systems using the *F*-measure, a commonly used metric in information retrieval and NLP. The *F*-measure combines precision and recall into a single score and is defined as the harmonic mean of precision and recall


(2) \begin{equation*} F-\mathrm{measure}=\frac{2\times \mathrm{Precision}\times \mathrm{Recall}}{\mathrm{Precision}+\mathrm{Recall}} \end{equation*}

where precision is the number of true positive predictions divided by the total number of predicted positives, and recall is the number of true positive predictions divided by the total number of actual positives in the dataset. The *F*-measure ranges from 0 to 1, with higher scores indicating better performance.

## CHALLENGE RESULTS

Compared with general fields, biomedical text mining requires lots of specific domain knowledge, making it challenging. Students are encouraged to participate in the AI CUP challenge, which aims to promote the development of NLP techniques and is open to participants from all domains. The PPIE track received 439 participants in 390 teams, 30 of which kept improving their prediction models on the public leaderboard. A total of 23 teams submitted predictions to the private leaderboard in the final submission, and the baseline model was developed as a definition of minimum performance. The training and development sets were integrated into an *N*-gram regression model, which demonstrated 21.3% performance. To qualify for a reward, participants must perform better than the baseline, and only 17 of the 18 teams were rewarded. One industrial team was excluded from the reward list. These teams represent 11 universities and cover various disciplines, including computer science, bioinformatics, electrical engineering and English.

Table 7 summarizes the top 10 teams’ performances and methods, as described in their system reports. The highest performance was achieved by Team_2, with a 77.06% *F*-measure. Text mining practices use DL as a mainstream approach. All teams using CNNs, NNs or Bi-LSTMs (Teams_2, 4, 6, 9 and 10) performed over 13.0% better than the baseline model. There was no significant difference between CNN models and the NN system in terms of performance. However, LSTM and Bi-LSTM achieved 16.0% higher performance than either of those models. Based on the PPIE track, it appears that LSTM has good contextual memory ability. Meanwhile, BERT provides powerful capabilities for managing input context. Team_14 and Team_17 both used BioBERT [92] with ensemble prediction. Furthermore, their models combined post-processing steps to eliminate event candidates with speculative mentions, such as may or might. This strategy achieved results above 76.0%, demonstrating its effectiveness. Team_6 and Team_13 used BERT as the base and concatenate it with diverse input pre-processing. This system design resulted in lower performance and is more unstable due to inappropriate design. Team_1 used a larger PLM, XLNet [16]. The lower performance indicates that XLNet, which is trained in the general domain, cannot predict biomedical tasks effectively. Teams integrated practical NLP libraries, such as NLTK [105], Pandas [106] and Sklearn [107] in data processing beyond the major model architectures.

**Table 7:** Summary of top 10 team participation in the PPIE track

Rank	Team no.	Team leader	Institution	${\mathrm{F}}_1$ -measure	Approach
1	Team_14	Jong-Kang Lee	National Central University	0.7706507	BioBERT
2	Team_17	Jue-Ni Huang	National Central University	0.7673446	BioBERT
3	Team_12	Ling-Hsuan Ying	National Cheng Kung University	0.7541494	CNN
4	Team_6	Chung-Yi Chen	National Sun Yat-sen University	0.6996493	BERT, LSTM, TextCNN
5	Team_2	Yi-Feng Wu	National Kaohsiung University of Applied Sciences	0.5558588	LSTM
6	Team_9	Kai-Ru Jeng	National Chung Hsing University	0.5133226	Attention-Based Bi-LSTM
7	Team_1	Chung-Yuan Cheng	National Yang-Ming University	0.4362394	XLNet
8	Team_10	Po-Ju Li	National Kaohsiung University of Applied Sciences	0.3534194	CNN
9	Team_4	Hao-Yu Hsu	Chang Gung University	0.3506743	NN
10	Team_13	Bo-Ren Mau	National Kaohsiung First University of Science and Technology	0.350129	BERT

We evaluated the performance of several BioPLMs on the PEDD dataset in Table 8. Besides BioBERT, we tested five other models: SciBERT [108], BlueBERT [95], PubMedBERT [109], BioRoBERTa [110] and CODER [111]. BioPLM comes in different versions, such as base and large. In order to enable more users to conduct testing, we primarily used the base version for experiments. We were using the Hugging Face package to train the model, with the hyperparameters set to a max_seq_length of 256, a per_device_train_batch_size of 8, a learning_rate of 5e−6 and a num_train_epochs of 25. The input for the PLM model is the raw CSV file. There are two columns in the input file: one with sentences and tagged normalized protein NE pairs and another with labels corresponding to the relation type. Take the following sentence as an instance:

**Table 8:** Performance of BioPLM system on the PEDD dataset

System	${\mathrm{F}}_1$ -measure
BioBERT	0.7602834
SciBERT	0.7525461
BlueBERT	0.7559562
PubMedBERT	0.7613945
BioRoBERTa	0.7633771
CODER	0.7578541

Raw sentence: ‘Grb2-associated regulator of Erk/MAPK1 (GAREM) is an adaptor molecule in the EGF-mediated signaling pathway.’

Preprocessed sentence: ‘@PROTEIN1$ is an adaptor molecule in the @PROTEIN2$-mediated signaling pathway.’

No other preprocessing strategies were applied in our experiments.

The process of converting a general-domain PLM into a BioPLM requires thorough pre-training. For instance, BioBERT and BlueBERT utilize weights and vocabulary from BERT for initialization. BioBERT is pre-trained on PubMed abstracts and PMC full-text articles, making it a useful model for biomedical text mining despite its smaller size. BlueBERT [95] is pre-trained on over 4000 million words of PubMed abstracts and over 500 million words of MIMIC-III clinical notes. Beltagy *et al.* [108] customized the vocabulary list and use the original BERT code to achieve this. They employ SentencePiece [112, 113] to create the list, of which only 42% of tokens overlap with BERT. SciBERT is pre-trained on a random sample of 1.14 million papers from Semantic Scholar [114], 82% of which are biomedical papers.

PubMedBERT is pre-trained on the domain-specific dataset (14 million PubMed abstracts, 3.2 billion words, 21 GB) from scratch, which is considered more effective than mixed-domain pre-training (such as BioBERT, BlueBERT and SciBERT) [109]. Pre-training on in-domain vocabulary has the benefit of training models with complete biomedical words rather than fragmented sub-words. Using PubMedBERT, the term ‘cardiomyocyte’ is considered a single medical term, while it is broken into five parts (cardiomyocyte) by BERT (BioBERT and BlueBERT behave similarly), and into two parts (cardiomyocyte) by SciBERT. The inclusion of in-domain pre-training data in model compilation is beneficial, as out-of-domain data can introduce noise to downstream tasks. PubMedBERT outperforms the aforementioned PLMs in several BioNLP tasks, including NER tasks, RE tasks (such as ChemProt, DDI and GAD) and the QA task (BioASQ). Gu *et al.* introduce not only PubMedBERT, but also a new benchmark called the Biomedical Language Understanding & Reasoning Benchmark (BLURB) compared with GLUE to enhance biomedical applications and explore new BioPLMs for pretraining.

BioRoBERTa [110] is derived from RoBERTa checkpoints through random initialization. The highest-performing BioRoBERTa model uses PubMed abstracts (22 million abstracts, 4.2 billion words, 27 GB), PMC full-text articles (3.4 million articles, 9.6 billion words, 60 GB), and MIMIC-III physician notes (0.5 billion words, 3.3 GB) for continual pretraining, along with a set of domain-specific vocabulary. To create the vocabulary, 50 000 sub-word units are learned from the PubMed pre-training corpus and the original RoBERTa general domain dictionary, using byte pair encoding (BPE) [112, 115, 116]. BioRoBERTa outperforms both BioBERT and SciBERT on most BioNLP tasks, largely due to its domain-specific vocabulary. CODER [111] is based on PLMs that utilize knowledge graph contrastive learning. The UMLS Metathesaurus, one of three UMLS Knowledge Sources, is used to incorporate biomedical terms and codes from various lexicon resources, as well as relations and attributes. CODER uses the UMLS Metathesaurus, which contains 4.27 million concepts, 15.48 million terms and 87.89 million relations.

According to Table 8, BioRoBERTa outperforms PubMedBERT, BioBERT, CODER, BlueBERT and SciBERT in the PEDD dataset. All models achieve scores above 0.75, demonstrating the strength of PLMs. Although there is a slight gap between the highest and lowest performing systems, the difference is only around 0.0108, indicating that they are relatively close in performance. Incorporating a domain-specific vocabulary for the PEDD dataset could further enhance the performance of these models. It is noteworthy that the gap between the IAA of the PEDD dataset (87.1% for relation types) and the performance of all systems is >10%. While BioPLMs outperform most participants, they still have room for improvement, as IAA is regarded as the upper limit of system performance [117–119]. Effective feature selection and comprehensive error analyses are potential strategies to improve the performance of these systems. In addition, the PEDD dataset has some relation types that are not adequately represented, reflecting the distribution of data in real-world bio-literature. To achieve better performance, some systems may ignore minor relation types, raising concerns about their robustness. Therefore, a comprehensive language model that addresses these issues without being limited by the amount of data is needed in the future.

## CONCLUSION

This paper presents a comprehensive review of the current state of biomedical RE datasets and systems. Moreover, the proposed PEDD dataset offers several distinct advantages compared with other existing PPI datasets, such as AIMed, LLL and Bioinfer. PEDD encompasses a larger number of documents, including more recent literature. While previous PPI datasets primarily focused on binary classification, PEDD goes a step further by defining finer-grained relation types. This granularity allows users to analyze context-specific categories with greater precision. The highest *F*-measure achieved by BioBERT-based models is an impressive 77.0%, while the recently introduced BioRoBERTa sets a new benchmark with an *F*-measure of 76.3%, demonstrating the potential of advanced BioPLMs. Here, we set the 76.3% *F*-measure of the best-performing model, BioRoBERTa, as the baseline for the PEDD dataset. However, there is still substantial room for improvement to reach the upper bound of performance. Notably, transformer-based models are the most commonly used approach for solving PPIE tasks, as evidenced by the participant systems we list. We expect that the PEDD dataset will contribute significantly to future BioNLP research and provide a valuable resource for training and testing advanced RE models.

Overall, this work highlights the significant potential of ML approaches for improving our understanding of complex biological systems and driving progress in the field of biomedical research. In fact, promoting the PEDD dataset only touches upon the fundamental issues in the field of RE. Real-world data often exhibit intrinsic complexities, such as data scarcity, domain shifts and diverse text structures. Researchers can address some of these limitations by thoughtfully integrating multiple models. For example, in the case of PEDD, which currently consists of abstracts, researchers can attempt to fine-tune the abstract-derived model using full-text data when applying it to full-length articles. Alternatively, they can employ a hybrid or ensemble system architecture by combining the existing abstract-trained model with pre-trained models based on full-text data to adapt to larger text scales. In terms of data preprocessing, relevant full-text information or distinct features can be extracted and integrated into the model. Moreover, when data prove insufficient for specific strategies in a target domain, the combination of publicly available domain-specific datasets with the creation of ideal validation datasets of the required scale may provide a potential solution. Finally, techniques such as few-shot learning, data augmentation and the incorporation of external knowledge sources are crucial for developing systems capable of effectively handling long-tail relation types. Each RE problem offers a variety of possible solutions, and by permuting and combining available techniques, we can uncover the core issues more deeply in the future.

**Key Points**

- Our article presents a comprehensive and systematic review of the latest biomedical datasets, systems and competitions relevant to relation extraction, offering an indispensable reference for researchers and successors.
- We introduce PEDD, a groundbreaking biomedical PPIE corpus that comprises gene pairs and diverse relation types, including 12 positive classes with corresponding negative counterparts. PEDD sets a new standard for complexity and diversity in PPIE datasets, making it a valuable resource for advancing the field of BioNLP.
- The PEDD dataset enables researchers to develop and test practical system applications for modern research descriptions, providing a more realistic and accurate representation of the challenges and complexities of real-world biomedical data.

## Supplementary Material

ppi_accuracy_mod_bbae132

test_ppi_accuracy_bbae132

## Data Availability

The PEDD dataset is available at https://drive.google.com/drive/folders/1BeFkvjdDMPAvY0zdBd59JECZBC-kqpb7.

## Author Biographies

**Ming-Siang Huang** is now a Postdoctoral Research Fellow in the Department of Computer Science and Information Engineering, College of Information and Electrical Engineering, Asia University, Taichung, Taiwan.

**Jen-Chieh Han**, MS (National Central University), is a Research Assistant in the Intelligent Information Service Research Laboratory, National Central University, Taoyuan, Taiwan.

**Pei-Yen Lin**, MS (National Taiwan University), is a Research Assistant in the Institute of Information Science, Academia Sinica, Taipei, Taiwan.

**Yu-Ting You**, MS (National Yang Ming University), is a Research Assistant in the Institute of Information Science, Academia Sinica, Taipei, Taiwan.

**Richard Tzong-Han Tsai** is a Professor of Computer Science and Information Engineering at National Central University and a Research Fellow at the GIS Center of Academia Sinica. With a strong publication record in leading journals, such as *Briefings in Bioinformatics*, *Bioinformatics*, *Nucleic Acids Research* and the *Journal of Medical Internet Research*, Dr Tsai’s expertise in the field is also reflected by his recent accomplishments, having won the 2020–2023 BioASQ competitions and multiple BioCreative competitions.

**Wen-Lian Hsu** is an IEEE Fellow, Chair Professor of the Department of Computer Science and Information Engineering, Asia University, New Taipei City, Taiwan, and Pervasive AI Research Labs, Ministry of Science and Technology, Taiwan.

## References

[1] Jiang F , Jiang Y, Zhi H, et al. Artificial intelligence in healthcare: past, present and future. Stroke Vasc Neurol 2017;2(4):230–43. 29507784 10.1136/svn-2017-000101 PMC5829945

[2] Jensen PB , Jensen LJ, Brunak S. Mining electronic health records: towards better research applications and clinical care. Nat Rev Genet 2012;13(6):395–405. 22549152 10.1038/nrg3208

[3] Evans RS . Electronic health records: then, now, and in the future. Yearb Med Inform 2016;25(Suppl 01):S48–61. 10.15265/IYS-2016-s006 PMC5171496 27199197

[4] Rajkomar A , Oren E, Chen K, et al. Scalable and accurate deep learning with electronic health records. NPJ Digit Med 2018;1(1):1–10. 31304302 10.1038/s41746-018-0029-1 PMC6550175

[5] Hirschman L , Park JC, Tsujii J, et al. Accomplishments and challenges in literature data mining for biology. Bioinformatics 2002;18(12):1553–61. 12490438 10.1093/bioinformatics/18.12.1553

[6] Li C , Liakata M, Rebholz-Schuhmann D. Biological network extraction from scientific literature: state of the art and challenges. Brief Bioinform 2014;15(5):856–77. 23434632 10.1093/bib/bbt006

[7] Rosenbloom ST , Denny JC, Xu H, et al. Data from clinical notes: a perspective on the tension between structure and flexible documentation. J Am Med Inform Assoc 2011;18(2):181–6. 21233086 10.1136/jamia.2010.007237 PMC3116264

[8] Wang Y , Wang L, Rastegar-Mojarad M, et al. Clinical information extraction applications: a literature review. J Biomed Inform 2018;77:34–49. 29162496 10.1016/j.jbi.2017.11.011 PMC5771858

[9] Huang M-S , Lai P-T, Lin P-Y, et al. Biomedical named entity recognition and linking datasets: survey and our recent development. Brief Bioinform 2020;21(6):2219–38. 32602538 10.1093/bib/bbaa054

[10] Industrial Technology Research Institute . AIdea Artificial Intelligence Collaboration Platform Available at: https://aidea-web.tw.

[11] Deléger L , Bossy R, Chaix E, et al. Overview of the bacteria biotope task at BioNLP shared task 2016. In: Proceedings of the 4th BioNLP Shared Task Workshop, Stroudsburg, PA: ACL, 2016, pp. 12–22.

[12] Bossy R , Golik W, Ratkovic Z, et al. Overview of the gene regulation network and the bacteria biotope tasks in BioNLP'13 shared task. BMC Bioinformatics 2015;16(10):1–16. 26202448 10.1186/1471-2105-16-S10-S1 PMC4511173

[13] Ding J , Berleant D, Nettleton D, Wurtele E. Mining MEDLINE: abstracts, sentences, or phrases? In: Biocomputing 2002. Kauai, Hawaii, USA: World Scientific, 2001, 326–37. 10.1142/9789812799623_0031 11928487

[14] Nédellec C . Learning language in logic-genic interaction extraction challenge. In: 4th Learning Language in Logic Workshop (LLL05). Born, Germany: ACM-Association for Computing Machinery, 2005.

[15] Bunescu R , Ge R, Kate RJ, et al. Comparative experiments on learning information extractors for proteins and their interactions. Artif Intell Med 2005;33(2):139–55. 15811782 10.1016/j.artmed.2004.07.016

[16] Pyysalo S , Ginter F, Heimonen J, et al. BioInfer: a corpus for information extraction in the biomedical domain. BMC Bioinformatics 2007;8(1):1–24. 17291334 10.1186/1471-2105-8-50 PMC1808065

[17] Fundel K , Küffner R, Zimmer R. RelEx—relation extraction using dependency parse trees. Bioinformatics 2007;23(3):365–71. 17142812 10.1093/bioinformatics/btl616

[18] Segura-Bedmar I , Martínez Fernández P, Sánchez Cisneros D. The 1st DDIExtraction-2011 Challenge Task: Extraction of Drug-Drug Interactions from biomedical texts. In: Proceedings of the 1st Challenge Task on Drug–drug Interaction Extraction, huelva spain, vol. 761, 2011, pp. 1–9.

[19] Herrero-Zazo M , Segura-Bedmar I, Martínez P, Declerck T. The DDI corpus: an annotated corpus with pharmacological substances and drug–drug interactions. J Biomed Inform 2013;46(5):914–20. 23906817 10.1016/j.jbi.2013.07.011

[20] Segura , Bedmar I, Martínez P, Herrero Zazo M. SemEval-2013 Task 9: Extraction of Drug-Drug Interactions from Biomedical Texts (DDIExtraction 2013). In: Proceedings of Semeval, Atlanta, GA, 2013, pp. 341–50.

[21] Gurulingappa H , Rajput AM, Roberts A, et al. Development of a benchmark corpus to support the automatic extraction of drug-related adverse effects from medical case reports. J Biomed Inform 2012;45(5):885–92. 22554702 10.1016/j.jbi.2012.04.008

[22] Gottlieb A , Stein GY, Ruppin E, Sharan R. PREDICT: a method for inferring novel drug indications with application to personalized medicine. Mol Syst Biol 2011;7(1):496. 21654673 10.1038/msb.2011.26 PMC3159979

[23] Wang W , Yang S, Zhang X, Li J. Drug repositioning by integrating target information through a heterogeneous network model. Bioinformatics 2014;30(20):2923–30. 24974205 10.1093/bioinformatics/btu403 PMC4184255

[24] Liang X , Zhang P, Yan L, et al. LRSSL: predict and interpret drug–disease associations based on data integration using sparse subspace learning. Bioinformatics 2017;33(8):1187–96. 28096083 10.1093/bioinformatics/btw770

[25] Wang A , Singh A, Michael J, et al. GLUE: a multi-task benchmark and analysis platform for natural language understanding. In: International Conference on Learning Representations, 2019. New Orleans, USA. ICLR.

[26] Huang H-Y , Lin YCD, Cui S, et al. miRTarBase update 2022: an informative resource for experimentally validated miRNA–target interactions. Nucleic Acids Res 2022;50(D1):D222–30. 34850920 10.1093/nar/gkab1079 PMC8728135

[27] Bodenreider O . The unified medical language system (UMLS): integrating biomedical terminology. Nucleic Acids Res 2004;32:D267–70. 14681409 10.1093/nar/gkh061 PMC308795

[28] Wishart DS , Knox C, Guo AC, et al. DrugBank: a knowledgebase for drugs, drug actions and drug targets. Nucleic Acids Res 2008;36:D901–6. 18048412 10.1093/nar/gkm958 PMC2238889

[29] Hamosh A , Scott AF, Amberger JS, et al. Online Mendelian inheritance in man (OMIM), a knowledgebase of human genes and genetic disorders. Nucleic Acids Res 2005;33:D514–7. 15608251 10.1093/nar/gki033 PMC539987

[30] Yang L , Agarwal P. Systematic drug repositioning based on clinical side-effects. PLoS One 2011;6(12):e28025. 22205936 10.1371/journal.pone.0028025 PMC3244383

[31] Campillos M , Kuhn M, Gavin A-C, et al. Drug target identification using side-effect similarity. Science 2008;321(5886):263–6. 18621671 10.1126/science.1158140

[32] Altman RB . PharmGKB: a logical home for knowledge relating genotype to drug response phenotype. Nat Genet 2007;39(4):426–6. 17392795 10.1038/ng0407-426 PMC3203536

[33] Luo L , Lai P-T, Wei C-H, et al. BioRED: a rich biomedical relation extraction dataset. Brief Bioinform 2022;23:bbac282. 35849818 10.1093/bib/bbac282 PMC9487702

[34] Krallinger M , Leitner F, Rodriguez-Penagos C, Valencia A. Overview of the protein-protein interaction annotation extraction task of BioCreative II. Genome Biol 2008;9(2):S4–19. 10.1186/gb-2008-9-s2-s4 PMC2559988 18834495

[35] Leitner F , Mardis SA, Krallinger M, et al. An overview of BioCreative II.5. IEEE/ACM Trans Comput Biol Bioinform 2010;7(3):385–99. 20704011 10.1109/tcbb.2010.61

[36] Li J , Sun Y, Johnson RJ, et al. BioCreative V CDR task corpus: a resource for chemical disease relation extraction. In: Database: the journal of biological databases and curation, 2016, baw068. 10.1093/database/baw068 PMC4860626 27161011

[37] Krallinger M , Rabal O, Akhondi SA, et al. Overview of the BioCreative VI chemical-protein interaction Track. In: Proceedings of the Sixth BioCreative Challenge Evaluation Workshop, Bethesda, MD USA: BioCreative, 2017, 142–7.

[38] Islamaj , Doğan R, Kim S, Chatr-aryamontri A, et al. Overview of the BioCreative VI precision medicine track: mining protein interactions and mutations for precision medicine. Database, 2019. 10.1093/database/bay147. PMC6348314 30689846

[39] Kerrien S , Aranda B, Breuza L, et al. The IntAct molecular interaction database in 2012. Nucleic Acids Res 2012;40(D1):D841–6. 22121220 10.1093/nar/gkr1088 PMC3245075

[40] Miranda A , Mehryary F, Luoma J, et al. Overview of DrugProt BioCreative VII track: quality evaluation and large scale text mining of drug-gene/protein relations. In: Proceedings of the Seventh BioCreative Challenge Evaluation Workshop, BioCreative, 2021, 11–21.

[41] Kim J-D , Ohta T, Pyysalo S, et al. Overview of BioNLP’09 shared task on event extraction. In: Proceedings of the BioNLP 2009 Workshop Companion Volume for Shared Task, Boulder, Colorado: Association for Computational Linguistics, 2009, 1–9.

[42] Kim J-D , Wang Y, Takagi T, Yonezawa A. Overview of Genia event task in BioNLP shared task 2011. In: Proceedings of BioNLP Shared Task 2011 Workshop, Portland, Oregon, USA: Association for Computational Linguistics, 2011, 7–15.

[43] Kim J-D , Ohta T, Tsujii JI. Corpus annotation for mining biomedical events from literature. BMC Bioinformatics 2008;9:1–25. 18182099 10.1186/1471-2105-9-10 PMC2267702

[44] Pyysalo S , Ohta T, Rak R, et al. Overview of the ID, EPI and REL tasks of BioNLP Shared Task 2011. In: BMC Bioinformatics. Springer, 2012;13:1–26. 10.1186/1471-2105-13-S11-S2 PMC3384257 22759456

[45] Nédellec C , Bossy R, Kim J-D, et al. Overview of BioNLP shared task 2013. In: Proceedings of the BioNLP Shared Task 2013 Workshop, Sofia, Bulgaria: Association for Computational Linguistics, 2013, 1–7.

[46] Kim J-D , Wang Y, Yasunori Y. The Genia event extraction shared task, 2013 edition - overview. In: Proceedings of the BioNLP Shared Task 2013 Workshop, Sofia, Bulgaria: Association for Computational Linguistics, 2013, 8–15.

[47] Pyysalo S , Ohta T, Ananiadou S. Overview of the cancer genetics (CG) task of BioNLP Shared Task 2013. In: Proceedings of the BioNLP Shared Task 2013 Workshop, Sofia, Bulgaria: Association for Computational Linguistics, 2013, 58–66.

[48] Ohta T , Rak R, Rowley A, et al. Overview of the pathway curation (PC) task of BioNLP shared task 2013. In: Proceedings of the BioNLP Shared Task 2013 Workshop, Sofia, Bulgaria: Association for Computational Linguistics, 2013, 67–75.

[49] Kim J-J , Han X, Lee VK, Schuhmann DR. GRO task: populating the gene regulation ontology with events and relations. In: Proceedings of the BioNLP Shared Task 2013 Workshop, Sofia, Bulgaria: Association for Computational Linguistics, 2013, 50-7.

[50] Bossy R , Bessières P, Nédellec C. BioNLP shared task 2013–an overview of the genic regulation network task. In: Proceedings of the BioNLP Shared Task 2013 Workshop, Sofia, Bulgaria: Association for Computational Linguistics, 2013, 153–60.

[51] Uzuner Ö , South BR, Shen S, DuVall SL. 2010 i2b2/VA challenge on concepts, assertions, and relations in clinical text. J Am Med Inform Assoc 2011;18(5):552–6. 21685143 10.1136/amiajnl-2011-000203 PMC3168320

[52] Henry S , Buchan K, Filannino M, et al. 2018 n2c2 shared task on adverse drug events and medication extraction in electronic health records. J Am Med Inform Assoc 2020;27(1):3–12. 31584655 10.1093/jamia/ocz166 PMC7489085

[53] Johnson AE , Pollard TJ, Shen L, et al. MIMIC-III, a freely accessible critical care database. Sci Data 2016;3(1):1–9. 10.1038/sdata.2016.35 PMC4878278 27219127

[54] Jagannatha A , Liu F, Liu W, Yu H. Overview of the first natural language processing challenge for extracting medication, indication, and adverse drug events from electronic health record notes (MADE 1.0). Drug Saf 2019;42(1):99–111. 30649735 10.1007/s40264-018-0762-z PMC6860017

[55] Doughty E , Kertesz-Farkas A, Bodenreider O, et al. Toward an automatic method for extracting cancer-and other disease-related point mutations from the biomedical literature. Bioinformatics 2011;27(3):408–15. 21138947 10.1093/bioinformatics/btq667 PMC3031038

[56] Pyysalo S , Ohta T, Miwa M, et al. Event extraction across multiple levels of biological organization. Bioinformatics 2012;28(18):i575–81. 22962484 10.1093/bioinformatics/bts407 PMC3436834

[57] Van Mulligen , Fourrier-Reglat A, Gurwitz D, et al. The EU-ADR corpus: annotated drugs, diseases, targets, and their relationships. J Biomed Inform 2012;45(5):879–84. 22554700 10.1016/j.jbi.2012.04.004

[58] Bravo À , Piñero J, Queralt-Rosinach N, et al. Extraction of relations between genes and diseases from text and large-scale data analysis: implications for translational research. BMC Bioinformatics 2015;16(1):1–17. 25886734 10.1186/s12859-015-0472-9 PMC4466840

[59] Alnazzawi N , Thompson P, Batista-Navarro R, Ananiadou S. Using text mining techniques to extract phenotypic information from the PhenoCHF corpus. BMC Med Inform Decis Mak 2015;15(2):1–10. 26099853 10.1186/1472-6947-15-S2-S3 PMC4474585

[60] Lee K , Lee S, Park S, et al. BRONCO: Biomedical entity relation ONcology COrpus for extracting gene-variant-disease-drug relations. Database 2016;2016:baw043. 10.1093/database/baw043 PMC4830473 27074804

[61] Peng N , Poon H, Quirk C, et al. Cross-sentence N-ary relation extraction with graph LSTMs. TACL 2017;5:101–15.

[62] Lai P-T , Lu WL, Kuo TR, et al. Using a large margin context-aware convolutional neural network to automatically extract disease-disease association from literature: comparative analytic study. JMIR Med Inform 2019;7(4):e14502. 31769759 10.2196/14502 PMC6913619

[63] Su J , Wu Y, Ting H-F, et al. RENET2: high-performance full-text gene–disease relation extraction with iterative training data expansion. NAR Genom Bioinform 2021;3:lqab062. 34235433 10.1093/nargab/lqab062 PMC8256824

[64] Wu Y , Luo R, Leung HC, et al. RENET: a deep learning approach for extracting gene-disease associations from literature. In: International Conference on Research in Computational Molecular Biology, Washington, DC, USA, Vol. 23. Springer, 2019, 272–84.

[65] Pyysalo S , Airola A, Heimonen J, et al. Comparative analysis of five protein–protein interaction corpora. BMC Bioinformatics 2008;9(3):1–11. 18426551 10.1186/1471-2105-9-S3-S6 PMC2349296

[66] Blaschke C , Andrade MA, Ouzounis CA, Valencia A. Automatic extraction of biological information from scientific text: protein–protein interactions. ISMB 1999;7:60–7. 10786287

[67] Ono T , Hishigaki H, Tanigami A, Takagi T. Automated extraction of information on protein–protein interactions from the biological literature. Bioinformatics 2001;17(2):155–61. 11238071 10.1093/bioinformatics/17.2.155

[68] Daraselia N , Yuryev A, Egorov S, et al. Extracting human protein interactions from MEDLINE using a full-sentence parser. Bioinformatics 2004;20(5):604–11. 15033866 10.1093/bioinformatics/btg452

[69] Blaschke C , Valencia A. The frame-based module of the SUISEKI information extraction system. IEEE Intell Syst 2002;17(2):14–20.

[70] Yakushiji A , Miyao Y, Tateisi Y, Tsujii J. Biomedical information extraction with predicate-argument structure patterns. In: Proceedings of the First International Symposium on Semantic Mining in Biomedicine (SMBM), Hinxton, UK: European Bioinformatics Institute. 2005, 93–6.

[71] Huang M , Zhu X, Hao Y, et al. Discovering patterns to extract protein–protein interactions from full texts. Bioinformatics 2004;20(18):3604–12. 15284092 10.1093/bioinformatics/bth451

[72] Mooney R . Relational learning of pattern-match rules for information extraction. In: Proceedings of the Sixteenth National Conference on Artificial Intelligence, Vol. 328. 1999, 334.

[73] Song M , Kim WC, Lee D, et al. PKDE4J: entity and relation extraction for public knowledge discovery. J Biomed Inform 2015;57:320–32. 26277115 10.1016/j.jbi.2015.08.008

[74] Sun L , Korhonen A. Improving verb clustering with automatically acquired selectional preferences. In: Proceedings of the 2009 Conference on Empirical Methods in Natural Language Processing: Volume 2-Volume 2 (Association for Computational Linguistics), 2009, 638–47.

[75] Vapnik VN . An overview of statistical learning theory. IEEE Trans Neural Netw 1999;10(5):988–99. 18252602 10.1109/72.788640

[76] Joachims T . Text categorization with support vector machines: Learning with many relevant features. In: European Conference on Machine Learning. Berlin, Heidelberg: Springer, 1998, 137–42.

[77] Mooney R , Bunescu R. Subsequence kernels for relation extraction. In: Proceedings of the Advances in Neural Information Processing Systems, Cambridge, MA, USA. MIT Press, 2005;171-8.

[78] Airola A , Pyysalo S, Björne J, et al. All-paths graph kernel for protein-protein interaction extraction with evaluation of cross-corpus learning. BMC Bioinformatics 2008;9(11):1–12. 19025688 10.1186/1471-2105-9-S11-S2 PMC2586751

[79] Miwa M , Sætre R, Miyao Y, Tsujii JI. A rich feature vector for protein-protein interaction extraction from multiple corpora. In: Proceedings of the 2009 Conference on Empirical Methods in Natural Language Processing, Volume 1. Singapore: Association for Computational Linguistics, 2009, 121–30.

[80] Tikk D , Thomas P, Palaga P, et al. A comprehensive benchmark of kernel methods to extract protein–protein interactions from literature. PLoS Comput Biol 2010;6:e1000837, 1–19. 20617200 10.1371/journal.pcbi.1000837 PMC2895635

[81] Giuliano C , Lavelli A, Romano L, Exploiting shallow linguistic information for relation extraction from biomedical literature. In: 11th Conference of the European Chapter of the Association for Computational Linguistics, Hindawi, London, UK, 2006, 401–8.

[82] LeCun Y , Bengio Y, Hinton G. Deep learning. Nature 2015;521(7553):436–44. 26017442 10.1038/nature14539

[83] Zeng D , Liu K, Lai S, et al. Relation classification via convolutional deep neural network. In: Proceedings of COLING 2014, the 25th International Conference on Computational Linguistics: Technical Papers, Dublin, Ireland: Dublin City University and Association for Computational Linguistics, 2014, 2335–44.

[84] Liu S , Tang B, Chen Q, Wang X. Drug-drug interaction extraction via convolutional neural networks. Comput Math Methods Med 2016;2016:6918381. 26941831 10.1155/2016/6918381 PMC4752975

[85] Gu J , Sun F, Qian L, Zhou G. Chemical-induced disease relation extraction via convolutional neural network. Database 2017:bax024. 10.1093/database/bax024 PMC5467558 28415073

[86] Peng Y , Lu Z. Deep learning for extracting protein-protein interactions from biomedical literature. In: Proceedings of the 2017 Workshop on Biomedical Natural Language Processing, Vancouver, Canada, 2017:29–38.

[87] Hsieh Y-L , Chang Y-C, Chang N-W, Hsu W-L. Identifying protein-protein interactions in biomedical literature using recurrent neural networks with long short-term memory. In: Proceedings of the Eighth International Joint Conference on Natural Language Processing (Volume 2: Short Papers), Taipei, Taiwan. Vol. 2, 2017, 240–5.

[88] Li F , Zhang M, Fu G, Ji D. A neural joint model for entity and relation extraction from biomedical text. BMC Bioinformatics 2017;18(1):1–11. 28359255 10.1186/s12859-017-1609-9 PMC5374588

[89] Lim S , Kang J. Chemical–gene relation extraction using recursive neural network. Database 2018;bay060. 10.1093/database/bay060 PMC6014134 29961818

[90] Zhang Y , Lin H, Yang Z, et al. A hybrid model based on neural networks for biomedical relation extraction. J Biomed Inform 2018;81:83–92. 29601989 10.1016/j.jbi.2018.03.011

[91] Devlin J , Chang M-W, Lee K, Toutanova K. Bert: pre-training of deep bidirectional transformers for language understanding. In: Proceedings of the 57th Annual Meeting of the Association for Computational Linguistics, Florence, Italy, 2019:4171–86.

[92] Lee J , Yoon W, Kim S, et al. BioBERT: a pre-trained biomedical language representation model for biomedical text mining. Bioinformatics 2020;36(4):1234–40. 31501885 10.1093/bioinformatics/btz682 PMC7703786

[93] Warikoo N , Chang Y-C, Hsu W-L. LBERT: lexically aware transformer-based bidirectional encoder representation model for learning universal bio-entity relations. Bioinformatics 2021;37(3):404–12. 32810217 10.1093/bioinformatics/btaa721

[94] Su P , Vijay-Shanker K. Investigation of BERT model on biomedical relation extraction based on revised fine-tuning mechanism. In: 2020 IEEE International Conference on Bioinformatics and Biomedicine (BIBM). IEEE, 2020, 2522–9.

[95] Peng Y , Yan S, Lu Z. Transfer learning in biomedical natural language processing: an evaluation of BERT and ELMo on ten benchmarking datasets. In: Proceedings of the 18th BioNLP Workshop and Shared Task, Florence, Italy, Association for Computational Linguistics, 2019:58–65.

[96] Lai P-T , Lu Z. BERT-GT: cross-sentence N-ary relation extraction with BERT and graph transformer. Bioinformatics 2020;36(24):5678–85. 10.1093/bioinformatics/btaa1087 PMC8023679 33416851

[97] Kalyan KS , Rajasekharan A, Sangeetha S. AMMU: a survey of transformer-based biomedical pretrained language models. J Biomed Inform 2022;126:103982. 34974190 10.1016/j.jbi.2021.103982

[98] Qureshi A , Thakur N, Monga I, et al. VIRmiRNA: a comprehensive resource for experimentally validated viral miRNAs and their targets. Database 2014;bau103. 10.1093/database/bau103 PMC4224276 25380780

[99] Bartel DP . Metazoan micrornas. Cell 2018;173(1):20–51. 29570994 10.1016/j.cell.2018.03.006 PMC6091663

[100] Maglott D , Ostell J, Pruitt KD, Tatusova T. Entrez gene: gene-centered information at NCBI. Nucleic Acids Res 2005;33:D54–8. 15608257 10.1093/nar/gki031 PMC539985

[101] Sole C , Larrea E, Manterola L, et al. Aberrant expression of MicroRNAs in B-cell lymphomas. Microrna 2016;5(2):87–105. 27568793

[102] McHugh ML . Interrater reliability: the kappa statistic. Biochem Med 2012;22(3):276–82. PMC3900052 23092060

[103] Altman DG . Practical statistics for medical research. London, Chapman and Hall, 1991.

[104] Sim J , Wright CC. The kappa statistic in reliability studies: use, interpretation, and sample size requirements. Phys Ther 2005;85(3):257–68. 15733050

[105] Bird S . NLTK: the natural language toolkit. In: Proceedings of the COLING/ACL 2006 Interactive Presentation Sessions, Sydney, Australia: Association for Computational Linguistics, 2006, 69–72.

[106] McKinney W . Data structures for statistical computing in python. In: Proceedings of the 9th Python in Science Conference, Austin, TX, Vol. 445. 2010, 51–6.

[107] Pedregosa F , Varoquaux G, Gramfort A, Michel V. Scikit-learn: machine learning in python. J Mach Learn Res 2011;12:2825–30.

[108] Beltagy I , Lo K, Cohan A. SciBERT: a pretrained language model for scientific text. In: Proceedings of the 2019 Conference on Empirical Methods in Natural Language Processing and the 9th International Joint Conference on Natural Language Processing (EMNLP-IJCNLP), Hong Kong, China: Association for Computational Linguistics, 2019, 3615–20.

[109] Gu Y , Tinn R, Cheng H, et al. Domain-specific language model pretraining for biomedical natural language processing. ACM Trans Comput Healthc 2021;3(1):1–23.

[110] Lewis P , Ott M, Du J, Stoyanov V. Pretrained language models for biomedical and clinical tasks: Understanding and extending the state-of-the-art. In: Proceedings of the 3rd Clinical Natural Language Processing Workshop, Association for Computational Linguistics, 2020, 146–57.

[111] Yuan Z , Zhao Z, Sun H, et al. CODER: knowledge-infused cross-lingual medical term embedding for term normalization. J Biomed Inform 2022;126:103983. 34990838 10.1016/j.jbi.2021.103983

[112] Sennrich R , Haddow B, Birch A. Neural machine translation of rare words with subword units. In: Proceedings of the 54th Annual Meeting of the Association for Computational Linguistics (Volume 1: Long Papers), Berlin, Germany: Association for Computational Linguistics, 2016: 1715–25.

[113] Kudo T . Subword regularization: improving neural network translation models with multiple subword candidates. In: Gurevych I, Miyao Y (eds) Proceedings of the 56th Annual Meeting of the Association for Computational Linguistics (Volume 1: Long Papers). Melbourne, Australia: Association for Computational Linguistics, 2018, 66–75.

[114] Ammar W , Groeneveld D, Bhagavatula C, et al. Construction of the literature graph in semantic scholar. In: Proceedings of the 2018 Conference of the North American Chapter of the Association for Computational Linguistics: Human Language Technologies, NAACL-HLT. New Orleans, Louisiana, USA: Association for Computational Linguistics, Vol. 3 (Industry Papers), 2018, 84–91.

[115] Gage P . A new algorithm for data compression. C Users J 1994;12(2):23–38.

[116] Radford A , Wu J, Child R, et al. Language models are unsupervised multitask learners. OpenAI Blog 2019;1(8):9.

[117] Gale WA , Church K, Yarowsky D. Estimating upper and lower bounds on the performance of word-sense disambiguation programs. In: Proceedings of the 30th Annual Meeting of the Association for Computational Linguistics. Association for Computational Linguistics, 1992, 249–56.

[118] Ormandjieva O , Hussain I, Kosseim L. Toward a text classification system for the quality assessment of software requirements written in natural language. In: Fourth International Workshop on Software Quality Assurance: in Conjunction with the 6th ESEC/FSE Joint Meeting. New York, US, Association for Computing Machinery, 2007, 39–45.

[119] Resnik P , Lin J. 11 evaluation of NLP systems. In: The Handbook of Computational Linguistics and Natural Language Processing, Wiley: Hoboken, NJ, USA, Vol. 57, 2010.

